# Differences in chronic obstructive pulmonary disease among US nursing home residents with heart failure according to sex and type of heart failure

**DOI:** 10.1111/crj.13698

**Published:** 2023-09-15

**Authors:** Seun Osundolire, Robert J. Goldberg, Kate L. Lapane

**Affiliations:** ^1^ Department of Population and Quantitative Health Sciences University of Massachusetts Medical School Worcester Massachusetts USA

**Keywords:** aging, chronic disease, comorbidities, epidemiology

## Abstract

**Background:**

Heart failure and chronic obstructive pulmonary disease (COPD) are leading cause of death throughout the world. Few recent studies have, however, examined possible sex and type of heart failure (HFpEF, HFrEF, and unspecified/other heart failure) differences in the prevalence of these chronic conditions among nursing home residents.

**Objectives:**

The aim of this study is to examine the magnitude of concomitant COPD and differences according to sex and heart failure type, in terms of the prevalence of COPD among nursing home residents with heart failure.

**Methods:**

The principal study outcomes were examined in a cross‐sectional study of 97 495 US nursing home residents with heart failure using the 2018 Minimum Data Set. The diagnoses of heart failure and COPD were operationalized through a review of nursing home admission, progress notes, and physical examination findings.

**Results:**

The average age of this study population was 81.3 ± 11.0 years, 67.3% were women, and 53.8% had COPD. A slightly higher prevalence of COPD was found among men than women. A higher proportion of unspecified heart failure type was found in both men and women, than reduced and preserved ejection fractions, respectively. In both men and women, there was a higher prevalence of COPD among those with various chronic conditions and current tobacco users.

**Conclusions:**

COPD is highly prevalent among medically complex middle‐aged and older nursing home residents with heart failure. Future research should focus on increasing our understanding of factors that influence the risk and optimal management of COPD and heart failure to improve the quality of life for nursing home residents.

## Graphical abstract

This study shows the sex and heart failure type differences in the sociodemographic and clinical characteristics of U. S nursing home residents with COPD and heart failure and addresses a gap in the current evidence and existing literature. In this study, 67.3% of residents were women, and 53.8% had COPD. A slightly higher prevalence of COPD was found among men than women.

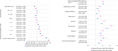

## 1 INTRODUCTION

Chronic obstructive pulmonary disease (COPD) contributes to more than 150 000 deaths annually in the United States and is the third leading cause of death globally with more than 3 million deaths attributed to this chronic condition on an annual basis.^1^ Another chronic condition of major public health and clinical importance is heart failure, which primarily affects older men and women. The combination of COPD and heart failure is associated with significant morbidity and mortality and reduced quality of life.^2^ The management of COPD is difficult and can be very challenging, particularly when this chronic pulmonary disease is diagnosed in conjunction with heart failure among older adults.

Previously, we reported on the overall prevalence of COPD among nursing home residents with heart failure.^3^ However, descriptive epidemiologic studies describing the extent to which the magnitude of COPD may differ according to sex and type of heart failure among older adults residing in nursing homes remain lacking, especially from a more generalizable population‐based perspective. Both COPD and heart failure have been historically underdiagnosed in women than in men.^4^ Data collected during the past decade, however, suggest that the prevalence of COPD may actually be similar among men and women^5^, ^6^, ^7^ and that quality of life may be more impaired in women than in men with COPD.^8^, ^9^, ^10^ Lastly, the type of heart failure, namely, whether due to preserved ejection fraction (HFpEF) or reduced ejection fraction (HFrEF) findings, may influence the magnitude, management, and impact of COPD.^11^ HFrEF is when the left ventricular ejection fraction is less than or equal to 40%, and HFpEF is when the left ventricular ejection fraction is greater than or equal to 50%.^12^ Unspecified/other heart failure type is the diagnoses of heart failure that was not distinguished by the clinician.

The objectives of this large observational study were to generate real‐world data to better understand possible differences in the prevalence of COPD according to sex and type of heart failure among nursing home residents in the United States with heart failure for purposes of identifying those groups that are most impacted by COPD and formulate effective clinical management practices and targeted interventions in this medically complex population.

## 2 METHODS

### 2.1 Data resources

To conduct our study, we linked three national datasets. The first database was the 2018 Minimum Data Set (MDS) Version 3.0. The MDS is a federally mandated geriatric clinical assessment required of all residents in Medicare and Medicaid certified nursing homes (>95% of all nursing homes in the United States), which contains a diverse population of older adults with multiple chronic conditions.^13^ The second database was the Medicare hospitalization Part A, MEDPAR file. This datafile includes uniform administrative and clinical elements obtained from health service claims for hospitalizations and skilled nursing facility admissions of all fee‐for‐service beneficiaries. The third database was the Medicare Master Beneficiary Summary File. This file contains information regarding the type of Medicare plan selected (entitlement on a monthly basis, Medicare Advantage, or Fee‐for‐Service Medicare) for all Medicare beneficiaries. This file allows for linkage of records across the MDS and MEDPAR files, while the Medicare Part A data include health service claims for hospitalizations and skilled nursing facility admissions of all fee‐for‐service beneficiaries that occurred between 2011 and 2018.

### 2.2 Study population

The study sample consisted of long‐term stay residents in US Medicare and Medicaid certified nursing homes in 2018. Included residents had at least one quarterly or annual MDS assessment performed in 2018. From these assessments and associated MEDPAR data, we identified residents with heart failure based on a validated algorithm from health claims data.^14^ Using the MEDPAR dataset, we identified and included residents having hospitalizations with primary heart failure related discharge ICD‐9 codes of 398.91 (rheumatic heart failure [congestive]), 402.01 or 402.11 or 402.91 (hypertensive heart failure), 404.01 or 404.03 or 404.11 or 404.13 or 404.91 or 404.93 (hypertensive heart failure and chronic kidney disease), or 428 (heart failure) or ICD‐10 heart failure codes I50 (heart failure), I09.81 (rheumatic heart failure), I11.0 (heart failure due to hypertension), I13.0 (heart failure due to hypertension with chronic kidney disease), or I13.2 (hypertensive heart and renal disease with both [congestive] heart failure and renal failure). Residents with at least one hospitalization before the index date (date of selected annual or quarterly MDS assessment in 2018) were included. We excluded residents who were classified as being in a comatose state, because these residents have vastly different care plans, and those in the nursing home for post‐acute care, which typically results in discharge after a few weeks.

We operationalized COPD using a validated health claims algorithm.^15^ Because the look‐back period for the validated algorithm for COPD was unspecified and COPD is a chronic, progressive disease, we used all the data available to the study team between 2011 and 2018. We used ICD‐9 codes 491 (chronic bronchitis), 492 (emphysema), or 496 (chronic airway obstruction, not elsewhere classified) for claims made before October 1, 2015, and used ICD‐10 codes J41 (simple chronic bronchitis), J43 (emphysema), or J44 (chronic obstructive pulmonary disease) for claims made after October 1, 2015. Residents with at least one MEDPAR claim with an indication of COPD before their MDS assessment date were classified as having COPD.

### 2.3 Stratification variables

The stratification variables were retrieved from either MDS data (sex) or claims data (heart failure type). Using claims data, residents with primary discharge codes ICD‐9 codes 428.2 or 428.4 or ICD‐10 codes I50.2 or I50.4 were classified as having HFrEF, those with ICD‐9 code 428.3 or ICD‐10 code I50.3 were classified as having HFpEF, and residents having ICD‐9‐CM codes 398.91, 402.01, 402.11, 402.91, 404.01, 404.03, 404.11, 404.13, 404.91, 404.93, 428.0, 428.1, or 428.9 or ICD‐10 codes I50.1, I50.8, I50.9, I11.0, I13.0, or I13.2 were classified as having unspecified heart failure.

### 2.4 Covariates

Selected covariates were derived from both the MDS and claims data, with the majority of study variables being taken from the MDS. Race/ethnicity was categorized as non‐Hispanic White, non‐Hispanic Black, Hispanic, and American Indian/Native Alaskan/Native Hawaiian/Other Pacific Islander/Multiple categorized as “others” due to the small number of residents in these racial/ethnic groups. We also considered several clinical characteristics (cognitive impairment and activities of daily living) and 19 comorbid conditions. Additional variables included were the social connectedness index, life expectancy and hospice care, use of oxygen, dyspnea (with exertion, when sitting at rest, and when lying flat), and tobacco use (of any kind). The social connectedness index variable involves three domains: (1) structure (connection to others via the existence of relationships and their roles), (2) function (sense of connection that results from actual or perceived support or inclusion), and (3) quality (sense of connection to others that is based on positive and negative qualities).^16^

The 19 comorbid conditions included were those that had been associated with COPD in previous research. These included nephropathy, neurological conditions (i.e., Alzheimer's disease and related dementias, stroke), gastroesophageal reflux disease, depression, anxiety, osteoporosis, hyperlipidemia, hypertension, anemia, obesity, cancer, cirrhosis, peripheral vascular disease, atrial fibrillation, pneumonia, coronary artery disease, and diabetes mellitus.^17^, ^18^, ^19^ Body mass index was categorized as ≤18.5 kg/m^2^, between ≥18.5 kg/m^2^ and ≤25 kg/m^2^, between >25 kg/m^2^ and <30 kg/m^2^, and ≥30 kg/m^2^. The comorbid conditions had a 7‐day look‐back period in the MDS. Nursing home staff documented conditions that required ongoing medication or therapy, a positive test, or procedure. Disease burden was described using a modified Charlson comorbidity index summary measure.

In terms of clinical characteristics, we used the cognitive function scale,^20^ which combines the Brief Interview for Mental Status and the Cognitive Performance Scale,^21^ while the resident's activities of daily living were determined using the MDS Activities of Daily Living (ADLs) Self‐Performance Hierarchy Scale. Scores between 0 and 4 indicate physical independence, whereas scores between 5 and 8 and between 9 and 12 indicate mild and moderate dependence, respectively.^22^

### 2.5 Statistical analysis

Descriptive analyses were conducted by sex and type of heart failure to show the distribution of the covariates by each stratification variable (i.e., sociodemographic, clinical characteristics, and comorbidities), and we estimated the prevalence of COPD by each level of key covariates. Because the sample size was large and *p*‐values are uninformative in this context, we considered absolute differences greater than 5% to be noteworthy. To account for different resident characteristics, we also estimated adjusted prevalence ratios derived from Poisson regression models stratified by sex and heart failure type. In these models, the outcome variable of interest was the binary indicator variable for COPD, and the independent variables were the covariates of interest. The adjusted estimates provided estimates of each variable independent of the other variables included in the models. We used the Poisson regression model with generalized estimating equations approach to account for the clustering of residents within nursing homes. Before implementing the stratified models, we conducted analyses to evaluate the potential for multicollinearity since this had been an issue in our previous work.^3^ Any variable whose variance inflation factor exceeded 2.5 was considered problematic, and only the Charlson comorbidity index was identified as being collinear. To address this issue, for each stratified Poisson regression analysis we created two multivariable adjusted models. The first model included all factors of interest with the exception of the Charlson comorbidity index, while the second model included all variables except for the individual disease diagnosis. Prevalence ratios and corresponding 95% confidence intervals were estimated from these models.

## 3 RESULTS

### 3.1 Study population

The sociodemographic, clinical characteristics, and comorbidities of the overall patient population of this study have been previously reported.^3^ There were 97 495 residents diagnosed with heart failure; among these residents, 53.8% had COPD. Approximately three quarters of our study sample were ≥75 years old, two‐thirds were women, and slightly more than three‐quarters were Non‐Hispanic Whites.

### 3.2 Stratified analysis by sex

Table 1 shows the sociodemographic, clinical characteristics, and comorbidities stratified by sex. Overall, 52.4% of women and 56.7% of men with heart failure also had concomitant COPD. Slightly more than one‐half of women were ≥ 85 years of age, whereas nearly one‐third of men were ≥85 years of age. The distribution of level of dependency in ADLs, level of cognitive impairment, and dementia diagnoses were similar in men and women. Mental health conditions were more commonly diagnosed in women than men and a greater proportion of men (23.4%) had reduced ejection fraction findings than women (16.6%). More than half of women and men had unspecified heart failure type. Most women and men were socially connected, and among men, 7.4% were current tobacco users, whereas 2.8% of women were current tobacco users (Table 1).

**TABLE 1:** Characteristics of long stay residents with heart failure living in US nursing homes in 2018 by sex.

Characteristics	Women (*n* = 65 609)	Men (*n* = 31 886)
	Percentage	
Age (years)		
<65	5.4	12.5
65–74	15.1	24.7
75–84	28.5	31.6
≥85	51.0	31.2
Race/ethnicity		
Non‐Hispanic White	79.3	73.4
Non‐Hispanic Black	14.7	18.6
Hispanic	4.1	6.0
American Indian/Native Alaskan or Hawaiian/Pacific Islander/multi	1.9	2.0
Activities of daily living		
Independent/supervised	20.2	25.0
Extensive limitations	58.6	58.4
Dependence	21.3	16.5
Cognitive impairment		
Cognitively intact	43.8	46.5
Mildly impaired	26.0	27.7
Moderately impaired	25.5	22.7
Severely impaired	4.7	3.2
Body mass index (kg/m^2^)		
<18.5	5.2	3.3
18.5 to 25	28.6	29.5
25 to <30	24.4	28.7
≥30	41.8	38.4
Co‐morbid conditions		
Alzheimer's disease	9.9	7.3
Other dementia	39.6	35.0
Coronary artery disease	32.3	39.9
Cerebrovascular disease	10.3	12.0
Renal insufficiency/end‐stage renal disease	29.3	35.7
Hypertension	88.5	87.0
Anxiety	35.8	24.9
Osteoporosis	13.2	3.4
Hyperlipidemia	53.7	58.8
Peripheral vascular disease	15.4	20.8
Atrial fibrillation	42.3	43.8
Anemia	41.0	39.3
Pneumonia	3.3	3.8
Depression	55.2	48.7
Cancer	5.2	7.3
Cirrhosis	0.7	1.5
Diabetes mellitus	47.3	54.6
Gastroesophageal reflux disease/ulcer	46.1	40.6
COPD	52.4	56.7
Charlson comorbidity index		
1–4	47.2	43.7
5	19.3	19.3
6–7	25.8	27.4
≥8	7.7	9.5
Social connectedness index = 5 (high)	89.1	86.5
Length of stay in nursing home greater than 1 year	34.5	27.1
Current tobacco user	2.8	7.4
On hospice	6.0	4.9
Life expectancy less than 6 months	5.1	4.3
Heart failure type		
Reduced ejection fraction	16.6	23.4
Preserved ejection fraction	25.8	17.6
Not specified	57.7	59.0
Any dyspnea	22.1	20.7
Short of breath with exertion	15.2	13.9
Short of breath sitting	5.5	5.1
Short of breath lying down	14.7	13.8
Use of oxygen	31.6	26.4

*Note*: Missing data: race/ethnicity (*n* = 1935), ADL (*n* = 23), BMI (*n* = 2852), Alzheimer's disease and dementia and pneumonia (*n* = 11), coronary artery disease (*n* = 13), stroke (*n* = 9), end stage renal disease and anemia (*n* = 14), hypertension (*n* = 15), anxiety and depression and GERD (*n* = 12), osteoporosis (*n* = 4), hyperlipidemia (*n* = 29), peripheral vascular disease (*n* = 5), atrial fibrillation (*n* = 8), cancer (*n* = 20), cirrhosis (*n* = 2), Charlson comorbidity index (*n* = 711), social index (*n* = 221), current tobacco use (*n* = 62), hospice (*n* = 162), life expectancy (*n* = 38), any shortness of breath (*n* = 47), shortness of breath with exertion (*n* = 42), sitting (*n* = 42), lying down (*n* = 64), and oxygen (*n* = 160).

Table 2 shows that in women, compared with residents 65–74 years of age, there was a lower prevalence of COPD across all other age groups, with the lowest prevalence observed in those ≥85 years old (adjusted prevalence ratio [aPR] in women: 0.74, 95% confidence interval [CI] 0.72, 0.75); similar results were noted in men (aPR in men [≥85 years old versus 65–74 years]: 0.80, 95% CI 0.78, 0.83). In both men and women, the prevalence of COPD was higher among those dependent in ADLs and the prevalence of COPD decreased with increasing levels of cognitive impairment. Depression and anxiety were associated with an increased prevalence of COPD in the two sexes (Figure S1), and both men and women with higher Charlson Comorbidity scores and who were current tobacco users had an increased prevalence of COPD relative to respective comparison groups (Table 2).

**TABLE 2:** Prevalence of chronic obstructive pulmonary disease (COPD) and association between sociodemographic, clinical characteristics, and comorbid conditions with COPD among long stay residents with heart failure living in US nursing homes in 2018 by sex.

	COPD prevalence by sex		Association between sociodemographic, clinical characteristics and comorbid conditions and COPD, by sex	
			Adjusted prevalence ratio (95% confidence interval)	
Characteristics	Women	Men	Women	Men
Age category (years)				
<65	67.3	61.1	0.95 (0.93–0.98)	0.91 (0.88–0.94)
65–74	66.7	64.5	Reference	Reference
75–84	59.6	58.5	0.95 (0.93–0.97)	0.96 (0.94–0.98)
≥85	42.6	47.0	0.74 (0.72–0.75)	0.80 (0.78–0.83)
Race/ethnicity				
Non‐Hispanic White	52.4	57.7	Reference	Reference
Non‐Hispanic Black	52.8	54.1	0.97 (0.95–0.99)	0.93 (0.90–0.95)
Hispanic	52.3	54.4	1.01 (0.96–1.05)	0.97 (0.93–1.02)
American Indian/Native Alaskan/Native Hawaiian/Pacific Islander	43.1	48.8	0.89 (0.83–0.96)	0.92 (0.85–1.00)
Activities of daily living				
Independent/supervised	54.1	59.8	Reference	Reference
Extensive limitations	51.3	55.4	1.00 (0.98–1.02)	0.98 (0.96–1.00)
Dependent	53.8	56.8	1.06 (1.03–1.08)	1.03 (1.00–1.06)
Cognitive impairment				
Intact	58.3	61.0	Reference	Reference
Mildly impaired	52.7	56.5	0.99 (0.97–1.01)	0.98 (0.96–1.01)
Moderately impaired	43.8	49.6	0.89 (0.87–0.91)	0.92 (0.89–0.94)
Severely impaired	41.9	46.3	0.86 (0.82–0.90)	0.84 (0.79–0.91)
Body mass index (kg/m^2^)				
<18.5	48.6	58.5	1.08 (1.04–1.12)	1.11 (1.05–1.17)
18.5 to 25.0	48.6	52.6	Reference	Reference
>25 to <30	48.6	53.9	1.01 (0.99–1.03)	1.00 (0.97–1.02)
≥30	59.7	61.9	1.10 (1.08–1.12)	1.07 (1.04–1.10)
Co‐morbid conditions				
Alzheimer's disease	45.4	50.9	0.96 (0.93–0.99)	0.98 (0.93–1.02)
Other dementia	47.8	52.1	0.96 (0.95–0.98)	0.95 (0.93–0.98)
Coronary artery disease	56.2	59.1	1.08 (1.07–1.10)	1.05 (1.03–1.08)
Cerebrovascular disease	49.7	54.5	0.93 (0.90–0.95)	0.96 (0.93–0.99)
Renal insufficiency/end‐stage renal disease	55.5	57.2	1.00 (0.98–1.01)	0.98 (0.96–1.00)
Hypertension	52.2	56.8	0.94 (0.92–0.96)	0.98 (0.95–1.01)
Anxiety	58.5	62.8	1.12 (1.10–1.14)	1.09 (1.06–1.11)
Osteoporosis	49.4	55.2	0.97 (0.95–1.00)	0.96 (0.91–1.02)
Hyperlipidemia	54.3	57.5	1.00 (0.99–1.02)	0.99 (0.97–1.01)
Peripheral vascular disease	55.3	60.9	1.00 (0.99–1.02)	1.05 (1.02–1.07)
Atrial fibrillation	52.5	57.4	1.03 (1.02–1.04)	1.03 (1.01–1.05)
Anemia	55.4	58.2	1.05 (1.03–1.06)	1.02 (1.00–1.04)
Pneumonia	65.8	66.4	1.22 (1.19–1.26)	1.16 (1.11–1.21)
Depression	56.1	59.3	1.07 (1.05–1.09)	1.03 (1.01–1.06)
Cancer	52.5	55.3	0.97 (0.94–1.00)	0.98 (0.95–1.02)
Cirrhosis	60.1	63.5	0.98 (0.90–1.05)	1.01 (0.94–1.08)
Gastroesophageal reflux disease/ulcer	56.9	61.5	1.10 (1.09–1.12)	1.11 (1.09–1.13)
Diabetes mellitus	57.7	58.9	1.05 (1.04–1.07)	1.02 (1.00–1.04)
Charlson comorbidity index				
1–4	47.8	53.9	Reference	Reference
5	53.6	56.9	1.09 (1.07–1.11)	1.05 (1.03–1.08)
6–7	57.0	59.1	1.13 (1.11–1.15)	1.08 (1.05–1.11)
≥8	62.3	62.6	1.20 (1.17–1.23)	1.13 (1.09–1.17)
Current tobacco user	86.5	79.5	1.45 (1.42–1.48)	1.36 (1.33–1.40)
Heart failure type				
Reduced ejection fraction	48.8	53.8	0.93 (0.91–0.95)	0.92 (0.89–0.95)
Preserved ejection fraction	53.1	58.3	Reference	Reference
Not specified	53.1	57.4	0.99 (0.98–1.01)	0.99 (0.96–1.01)
Any dyspnea	71.0	75.7	1.22 (1.19–1.24)	1.20 (1.18–1.22)
Short of breath with exertion	70.8	76.2	1.09 (1.06–1.12)	1.06 (1.04–1.08)
Short of breath sitting	73.6	80.1	0.96 (0.93–0.99)	0.95 (0.93–0.98)
Short of breath lying down	74.1	78.4	1.19 (1.16–1.22)	1.19 (1.17–1.22)
Use of oxygen	71.8	76.6	1.40 (1.37–1.42)	1.44 (1.42–1.46)

*Note*: Missing data: race/ethnicity (*n* = 1935), activities of daily living (*n* = 23), body mass index (*n* = 2852), Alzheimer's disease and dementia and pneumonia (*n* = 11), coronary artery disease (*n* = 13), stroke (*n* = 9), end stage renal disease and anemia (*n* = 14), hypertension (*n* = 15), anxiety and depression and gastroesophageal reflux disease/ulcer (*n* = 12), osteoporosis (*n* = 4), hyperlipidemia (*n* = 29), peripheral vascular disease (*n* = 5), atrial fibrillation (*n* = 8), cancer (*n* = 20), cirrhosis (*n* = 2), Charlson comorbidity index (*n* = 711), and current tobacco use (*n* = 62).

### 3.3 Stratified analysis by heart failure type

The prevalence of COPD was higher and similar among those who had preserved (54.4%) and unspecified (54.5%) heart failure types than among residents with reduced heart failure findings (50.9%). There was a higher proportion of residents who were aged ≥85 years, non‐Hispanic Whites, and obese in those who had preserved ejection fraction findings (Table 3).

**TABLE 3:** Characteristics of long stay residents with heart failure living in US nursing homes in 2018 by heart failure type.

	Heart failure type		
Characteristics	Reduced ejection fraction (*n* = 18 338)	Preserved ejection fraction (*n* = 22 502)	Other/unspecified (*n* = 56 655)
Age category (years)			
<65	7.6	6.2	8.3
65–74	18.4	16.2	19.0
75–84	30.6	28.4	29.6
≥85	43.5	49.2	43.1
Sex			
Women	59.3	75.1	66.8
Race/ethnicity			
Non‐Hispanic White	77.5	82.1	75.5
Non‐Hispanic Black	15.8	12.6	17.3
Hispanic	4.8	3.7	5.1
American Indian/Native Alaskan or Hawaiian/Pacific Islander/Multi	1.9	1.5	2.1
Activities of daily living			
Independent/supervised	24.6	20.0	21.5
Extensive limitations	56.8	59.4	58.8
Dependence	18.6	20.6	19.7
Cognitive impairment			
Cognitively intact	42.2	44.3	45.6
Mildly impaired	26.8	26.0	26.7
Moderately impaired	26.9	25.2	23.6
Severely impaired	4.1	4.6	4.1
Body mass index (kg/m^2^)			
<18.5	5.2	4.3	4.5
18.5 to 25	32.2	26.0	29.0
25 to <30	27.3	25.0	25.6
≥30	35.3	44.7	40.9
Co‐morbid conditions			
Alzheimer's disease	9.8	9.7	8.6
Other dementia	40.2	39.9	36.7
Coronary artery disease	39.2	31.5	34.7
Cerebrovascular disease	11.7	10.5	10.8
Renal insufficiency/end‐stage renal disease	28.5	29.3	33.1
Hypertension	86.9	86.7	88.9
Anxiety	30.4	34.5	31.9
Osteoporosis	9.3	11.4	9.7
Hyperlipidemia	56.1	53.7	55.8
Peripheral vascular disease	17.4	17.0	17.1
Atrial fibrillation	45.3	43.8	41.7
Anemia	37.4	40.7	41.4
Pneumonia	2.7	3.2	3.8
Depression	52.5	55.3	52.3
Cancer	5.8	6.0	5.9
Cirrhosis	1.0	1.0	1.0
Diabetes mellitus	47.6	51.9	51.0
Gastroesophageal reflux disease/ulcer	42.7	45.8	44.2
Chronic obstructive pulmonary disease	50.9	54.4	54.5
Charlson comorbidity index			
1–4	46.7	47.1	45.5
5	19.4	19.1	19.4
6–7	25.9	25.6	26.7
≥8	8.9	8.2	8.4
Social connectedness index = 5 (high)	87.8	87.9	88.5
Length of stay > 1 year	40.9	41.5	25.5
Current tobacco user	5.0	3.4	4.5
On hospice	5.6	5.7	5.6
Life expectancy < 6 months	4.7	5.0	4.8
Any dyspnea	18.7	22.4	22.3
Short of breath with exertion	13.0	15.3	15.2
Short of breath sitting	4.3	5.6	5.6
Short of breath lying down	11.9	15.1	15.0
Use of oxygen	24.0	31.7	31.1

Among nursing home residents with a reduced ejection fraction, factors associated with an increased prevalence of COPD included current tobacco use, underweight and obesity, dependency in ADLs, and several comorbid conditions (i.e., gastroesophageal reflux disease, pneumonia, anxiety, depression, anemia, diabetes mellitus, peripheral vascular disease, and coronary artery disease) (Table 4). Factors associated with a decreased frequency of diagnosed COPD among residents who had reduced ejection fraction findings included age, cognitive impairment, cerebrovascular disease, and racial/ethnic minorities (American Indian/Native Alaskan/Native Hawaiian/Pacific Islander/multi group, and non‐Hispanic Blacks). In comparison with those aged 65–74 years old, a lower prevalence of unspecified, reduced, and preserved ejection fraction, respectively, was found among those younger than 65 years, between 75 and 84 years, and ≥85 years old (Figure S2). Across all heart failure types, residents with mild, moderate, and severe cognitive impairment had a lower risk of COPD, compared to those with intact cognition. Residents with cerebrovascular disease were less likely to be diagnosed with COPD, compared to those without this condition (aPR: 0.91, 95% confidence interval [CI] 0.87, 0.96). With respect to race/ethnicity, American Indian/Native Alaskan/Native Hawaiian/Pacific Islander/multi group and non‐Hispanic Blacks had a lower prevalence of COPD relative to non‐Hispanic Whites.

**TABLE 4:** Prevalence of chronic obstructive pulmonary disease (COPD) and association between sociodemographic, clinical characteristics, and comorbid conditions and COPD among long stay residents with heart failure living in US nursing homes in 2018 by heart failure type.

	Heart failure type			Adjusted prevalence ratio (95% confidence interval)		
Characteristics	Reduced ejection fraction (*n* = 18 338)	Preserved ejection fraction (*n* = 22 502)	Other (*n* = 56 655)	Reduced ejection fraction	Preserved ejection fraction	Other/unspecified
Age category (years)						
<65	61.6	68.4	63.4	0.94 (0.90–0.99)	0.93 (0.89–0.98)	0.92 (0.90–0.94)
65–74	62.5	68.6	65.7	Reference	Reference	Reference
75–84	55.3	60.3	60.0	0.94 (0.91–0.97)	0.95 (0.92–0.98)	0.96 (0.94–0.98)
≥85	40.9	44.4	44.1	0.73 (0.70–0.76)	0.75 (0.73–0.78)	0.76 (0.74–0.78)
Sex						
Women	48.8	53.1	53.1	0.98 (0.95–1.01)	0.96 (0.94–0.99)	0.97 (0.96–0.99)
Men	53.8	58.3	57.4	Reference	Reference	Reference
Race/ethnicity						
Non‐Hispanic White	51.2	54.1	55.0	Reference	Reference	Reference
Non‐Hispanic Black	49.9	56.6	53.4	0.94 (0.90–0.98)	1.01 (0.97–1.04)	0.94 (0.92–0.96)
Hispanic	51.8	53.8	53.4	1.03 (0.96–1.10)	1.01 (0.94–1.08)	0.98 (0.94–1.02)
American Indian/Native Alaskan/Native Hawaiian/Pacific Islander	41.5	43.0	46.7	0.86 (0.75–0.99)	0.89 (0.79–1.00)	0.82 (0.86–0.99)
Activities of daily living						
Independent/supervised	52.8	56.4	57.4	Reference	Reference	Reference
Extensive limitations	49.8	53.1	53.3	1.01 (0.98–1.05)	1.00 (0.97–1.03)	0.99 (0.97–1.00)
Dependent	51.6	55.9	55.0	1.07 (1.02–1.12)	1.06 (1.02–1.10)	1.04 (1.02–1.07)
Cognitive impairment						
Intact	56.2	60.0	59.9	Reference	Reference	Reference
Mildly impaired	51.3	54.6	54.7	0.99 (0.95–1.02)	0.99 (0.96–1.02)	0.99 (0.97–1.01)
Moderately impaired	43.6	45.9	46.2	0.89 (0.85–0.93)	0.89 (0.85–0.92)	0.91 (0.88–0.93)
Severely impaired	40.6	45.2	42.8	0.81 (0.73–0.89)	0.90 (0.83–0.97)	0.84 (0.80–0.89)
Body mass index (kg/m^2^)						
<18.5	49.8	47.5	52.6	1.10 (1.03–1.18)	1.01 (0.94–1.08)	1.10 (1.06–1.15)
18.5 to 25.0	46.7	47.9	48.6	Reference	Reference	Reference
>25 to <30	48.9	49.7	51.4	1.00 (0.96–1.04)	0.99 (0.96–1.03)	1.01 (0.99–1.04)
≥30	56.2	61.5	61.1	1.05 (1.01–1.09)	1.10 (1.07–1.14)	1.10 (1.08–1.12)
Co‐morbid conditions						
Alzheimer's disease	45.7	48.0	46.8	0.98 (0.93–1.04)	0.98 (0.93–1.02)	0.95 (0.92–0.98)
Other dementia	47.1	49.5	49.6	0.98 (0.86–1.01)	0.96 (0.94–0.99)	0.96 (0.94–0.97)
Coronary artery disease	54.8	57.6	58.1	1.08 (1.05–1.12)	1.08 (1.05–1.11)	1.07 (1.05–1.08)
Cerebrovascular disease	48.0	51.3	52.7	0.91 (0.87–0.96)	0.93 (0.89–0.97)	0.95 (0.93–0.97)
Renal insufficiency/end stage renal disease	53.7	55.5	57.0	1.01 (0.98–1.04)	0.96 (0.94–0.99)	0.99 (0.98–1.01)
Hypertension	50.9	54.1	54.4	0.98 (0.93–1.02)	0.94 (0.91–0.98)	0.95 (0.93–0.97)
Anxiety	55.6	60.2	60.5	1.08 (1.04–1.11)	1.11 (1.08–1.14)	1.12 (1.10–1.14)
Osteoporosis	49.0	50.1	50.3	1.01 (0.96–1.06)	0.96 (0.92–1.00)	0.96 (0.93–0.98)
Hyperlipidemia	52.8	55.8	56.1	1.02 (0.99–1.15)	0.98 (0.96–1.01)	1.00 (0.98–1.02)
Peripheral vascular disease	54.8	57.0	58.6	1.03 (0.99–1.07)	1.01 (0.98–1.05)	1.03 (1.01–1.05)
Atrial fibrillation	51.1	54.1	55.2	1.02 (0.99–1.05)	1.02 (0.99–1.04)	1.04 (1.02–1.06)
Anemia	53.3	56.2	57.2	1.04 (1.01–1.07)	1.02 (0.99–1.04)	1.05 (1.03–1.07)
Pneumonia	65.7	68.1	65.3	1.27 (1.19–1.36)	1.22 (1.16–1.29)	1.18 (1.14–1.21)
Depression	54.2	57.7	57.8	1.07 (1.04–1.11)	1.06 (1.04–1.09)	1.05 (1.03–1.07)
Cancer	51.6	54.9	53.8	0.98 (0.92–1.04)	1.00 (0.95–1.05)	0.97 (0.94–1.00)
Cirrhosis	56.5	61.5	63.7	0.93 (0.82–1.06)	0.95 (0.85–1.06)	1.02 (0.96–1.09)
Gastroesophageal reflux disease/ulcer	55.4	58.7	59.0	1.11 (1.08–1.14)	1.11 (1.09–1.14)	1.10 (1.09–1.12)
Diabetes mellitus	55.0	59.1	58.7	1.04 (1.01–1.08)	1.04 (1.02–1.07)	1.04 (1.03–1.06)
Charlson comorbidity index						
1–4	46.7	51.1	50.1	Reference	Reference	Reference
5	51.6	55.3	55.5	1.09 (1.04–1.13)	1.06 (1.03–1.10)	1.09 (1.06–1.11)
6–7	54.6	57.8	58.7	1.13 (1.09–1.17)	1.08 (1.05–1.12)	1.12 (1.10–1.14)
≥8	61.2	61.0	63.4	1.24 (1.18–1.30)	1.11 (1.06–1.16)	1.19 (1.16–1.22)
Current tobacco user	80.5	86.1	82.3	1.44 (1.39–1.50)	1.39 (1.34–1.44)	1.39 (1.35–1.42)

*Note*: Missing data: race/ethnicity (*n* = 1935), activities of daily living (*n* = 23), body mass index (*n* = 2852), Alzheimer's disease and dementia and pneumonia (*n* = 11), coronary artery disease (*n* = 13), stroke (*n* = 9), end stage renal disease and anemia (*n* = 14), hypertension (*n* = 15), anxiety and depression and gastroesophageal reflux disease (*n* = 12), osteoporosis (*n* = 4), hyperlipidemia (*n* = 29), peripheral vascular disease (*n* = 5), atrial fibrillation (*n* = 8), cancer (*n* = 20), cirrhosis (*n* = 2), Charlson comorbidity index (*n* = 711), and current tobacco user (*n* = 62).

Among residents with preserved ejection fraction values, a relatively similar profile emerged with few differences noted. Nursing home residents with end stage renal disease and hypertension had a decreased prevalence of COPD in comparison to those without these conditions and women had a lower prevalence of COPD than men. For residents with unspecified heart failure type, the profile associated with COPD was similar to those who had other types of heart failure with a few exceptions. Hypertension and osteoporosis were associated with a lower prevalence of COPD, and peripheral vascular disease and atrial fibrillation were associated with an increased prevalence of COPD.

## 4 DISCUSSION

The present study was a follow‐up to our previous research on the descriptive epidemiology of COPD among US nursing home residents with heart failure.^3^ In the present investigation, COPD was highly prevalent, regardless of sex or type of heart failure. More than one‐half of men and women with heart failure were also diagnosed with COPD. In both men and women, factors associated with a greater prevalence of COPD were dependence on ADL, higher Charlson comorbidity index scores, and current tobacco use. The majority of nursing home residents had a preserved ejection fraction or unspecified heart failure type, and the frequency of COPD was higher and similar among those with preserved ejection fraction and unspecified heart failure type. Based on the type of heart failure present, factors associated with a greater prevalence of COPD were current tobacco use, obesity, peripheral vascular disease, atrial fibrillation, gastroesophageal reflux disease, and pneumonia.

### 4.1 Sex differences in the prevalence of COPD among nursing home residents with heart failure

The crude prevalence of COPD was slightly lower in women than in men in the present study. Despite women being considerably older than men, both sexes had relatively similar profiles of being medically complex with cognitive impairment, physical function limitations, and multiple comorbid conditions commonly noted. A study conducted by the Centers for Disease Control and Prevention showed that American women (5.6%) had a slightly higher prevalence of COPD than men (4.4%) in the general population in 2020.^23^ In a systematic review and meta‐analysis of 194 studies conducted in 58 countries (including the United States), the prevalence of COPD was 9.2% in men and 6.2% in women, with the highest prevalence of heart failure in women found in North America.^24^ In the general US population, COPD is becoming increasingly common among women, in part because they are less likely to quit smoking than men.^25^, ^26^, ^27^

Approximately 22% of women in developed countries and 9% of women in developing countries use tobacco, but the frequency of tobacco use among women in developing countries is predicted to more than double by 2025.^28^ Furthermore, results of a large longitudinal data study conducted in the United Kingdom, the United States, Canada, and Australia confirmed that women have a greater difficulty sustaining tobacco abstinence than men.^29^ Therefore, increases in the occurrence of COPD among women are expected in the future. To avoid underdiagnosis or misdiagnosis^30^ and for effective medical management, clinicians need to become well versed in these sex differences for the development of COPD.

We found that women less than 74 years of age had a higher prevalence of COPD than similarly aged men. The high prevalence of COPD among relatively younger women with heart failure living in the nursing home setting likely can be attributed to the increasing number of American women who used tobacco products over the past several decades.^30^ Further, women experience an increased dose‐dependent susceptibility to COPD because they have smaller lungs and airways than men.^31^ Women have also had an increased susceptibility to secondhand smoke,^32^ as well as hormonally mediated differences in tobacco metabolism.^33^, ^34^

Current treatment guidelines for nursing home residents with COPD have been implemented from studies that are disproportionately male‐centric.^35^ Our results showed a much greater burden of COPD associated with several comorbidities among women, so it remains important for treating clinicians to recognize the importance of sex‐specific approaches in their management practices.

### 4.2 Differences in the prevalence of COPD among nursing home residents according to type of heart failure

We found a higher prevalence of nursing home residents with unspecified heart failure type and heart failure with a preserved ejection fraction. The high prevalence of unspecified heart failure type was likely due to the lack of comprehensive cardiac work‐up by clinicians in the nursing home setting. This is somewhat surprising because echocardiography, an inexpensive, widely available, and noninvasive technique, is beneficial for diagnosing the type of heart failure and implementing tailored treatment strategies.

The high frequency of nursing home residents with a preserved ejection fraction observed in our study is consistent with the existing literature which has shown that heart failure with preserved ejection fraction is more common than heart failure with reduced ejection fraction among older adults.^36^, ^37^, ^38^

The prevalence of COPD was higher and similar among residents with preserved ejection fraction (54.4%) and unspecified heart failure type (54.5%) than those with reduced ejection fraction (50.9%). A retrospective study conducted using the Acute Decompensated Heart Failure National Registry (ADHERE) database consisting of 105 388 patient admissions for heart failure at 274 centers showed a higher prevalence of COPD in patients with preserved ejection fraction (31%), than the prevalence of COPD in patients with reduced ejection fraction (27%).^39^ Compared to the ADHERE study, the higher prevalence of COPD could be due to the older population found in our study. The average age of residents in our study was 81.3 years, while the average age of the patients in the ADHERE study was 72.2 years.

### 4.3 Association between comorbid conditions and COPD among nursing home residents with heart failure by sex

We found that in both men and women, comorbid conditions such as coronary artery disease, anemia, diabetes mellitus, anxiety, depression, pneumonia, and gastroesophageal reflux disease were associated with an increased frequency of COPD. These findings are consistent with prior studies throughout the world.^40^, ^41^, ^42^, ^43^, ^44^ In a study of more than 1.1 million men and women treated at 950 hospitals in 30 US states in 2012, the presence of several commonly diagnosed chronic conditions was associated with a higher risk for emergency department visits for COPD.^45^ Our recent study on COPD among US nursing home residents with heart failure showed that pneumonia was the most commonly diagnosed condition among the 19 comorbid conditions examined.^3^ Other studies have also found this pulmonary condition to be the most frequent comorbid condition and a key risk factor for COPD.^46^, ^47^ Our finding regarding the association between gastroesophageal reflux disease and COPD is consistent with previous research findings.^48^, ^49^ The high prevalence of multiple comorbidities among residents with COPD decreases quality of life leads to more frequent exacerbations and further complicates the management of this complex disease. Therefore, COPD should be seen as a multi‐system disorder affecting the cardiovascular, endocrine, musculoskeletal, and gastrointestinal systems, and a more holistic screening strategy and interventional procedures should be implemented while managing persons with these additional chronic conditions.

We also found that anxiety and depression were associated with an increased prevalence of COPD in both men and women. This finding is consistent with a systematic review of 81 studies published between 1968 and 2004 which showed that patients with COPD have a higher prevalence of anxiety and depression than the general population.^40^ While clinicians may suggest psychological (relaxation, cognitive behavioral therapy) and pharmacological interventions for anxiety and depression in patients with COPD,^50^ the extent to which these interventions are effective in nursing home residents is unknown. Further studies and guidelines remain needed to enhance screening for various mental health conditions and improve the management of anxiety and depression in nursing home residents with COPD.^50^

### 4.4 Association between comorbid conditions and COPD among nursing home residents with heart failure by heart failure type

Both heart failure and COPD share similar pathophysiological processes which require careful evaluation and interpretation of cardiac and pulmonary function tests. This is because of the presence of overlapping signs and symptoms, such as reduced exercise tolerance, exertional fatigue, and dyspnea, leading to misdiagnosis or inappropriate treatment.^51^

In our study, among those with reduced and preserved ejection fractions and unspecified heart failure type, co‐morbid conditions such as obesity, gastroesophageal reflux disease, pneumonia, anxiety, depression, anemia, diabetes mellitus, peripheral vascular disease, coronary artery disease, atrial fibrillation were associated with a high prevalence of COPD. Several studies that included patients with heart failure and COPD have consistently shown that these cardiovascular and non‐cardiovascular co‐morbid conditions are prevalent in older adults and play a significant role in the pathophysiology of heart failure with reduced or preserved ejection fractions.^36^, ^37^, ^52^, ^53^, ^54^

### 4.5 Study strengths and limitations

This study focused on nursing home residents with heart failure who are medically complex and often excluded from clinical research. It was conducted using a large national database of nursing home residents with a high prevalence of co‐occurring COPD and heart failure, which allowed us to conduct analyses further stratified by sex and heart failure type. In our study, a validated algorithm was used to define COPD and heart failure based on ICD codes,^15^, ^55^ rather than self‐reported diagnoses.

There are several limitations to our study that need to be considered in the interpretation of the study results. The residents with heart failure with mildly reduced ejection fraction (HFmrEF, i.e., left ventricular ejection fraction between 41% and 49%) were not examined in this study. The MDS does not collect information on pulse oximetry values, complete blood count, family history of heart failure or COPD, and genetic factors. The MDS did not have data available on forced expiratory volume, which can be informative on the forms and severity of COPD among nursing home residents.

## 5 CONCLUSION

Understanding differences in the prevalence of COPD in men and women and according to the type of heart failure in nursing home residents with heart failure is necessary for delivering effective medical and lifestyle interventions in this vulnerable and clinically complex population. There is a lack of evidence for the effective management of both COPD and heart failure among nursing home residents. The management of these co‐occurring chronic conditions is complex due to their overlapping pathophysiological processes, and few studies have designed effective management protocols for these conditions in the nursing home population. This study highlights the high prevalence and risk of COPD among women and older adults with heart failure.

## AUTHOR CONTRIBUTIONS

Seun Osundolire conceptualized and designed the study, wrote the original draft, and did formal analysis. Robert J. Goldberg revised original draft and edited the manuscript. Kate L. Lapane was involved in the methodology, formal analysis, and supervision of the study.

## ACKNOWLEDGEMENTS

None.

## CONFLICT OF INTEREST STATEMENT

Drs. Osundolire, Goldberg, and Lapane have no conflicts to report.

## ETHICS STATEMENT

The Institutional Review Board of the University of Massachusetts Chan Medical School approved this cross‐sectional study. The data accessed complied with relevant data protection and privacy regulations.

## Supporting information


**Figure S1.** Association between sociodemographic, clinical characteristics and comorbid conditions and COPD, stratified by sex.
**Figure S2:** Association between sociodemographic, clinical characteristics and comorbid conditions with COPD, stratified by heart failure type.

## Data Availability

The data that support the findings of this study are available on request from the corresponding author. The data are not publicly available due to privacy or ethical restrictions.
