# Supplementary material for: Differences in chronic obstructive pulmonary disease among US nursing home residents with heart failure according to sex and type of heart failure
Source: Clin Respir J. 2023 Sep 15;17(11):1130–44. doi: 10.1111/crj.13698 (PMC10632080; doi:10.1111/crj.13698)

**Supplementary Figure 1: Association between sociodemographic, clinical characteristics and comorbid conditions and COPD, stratified by sex**

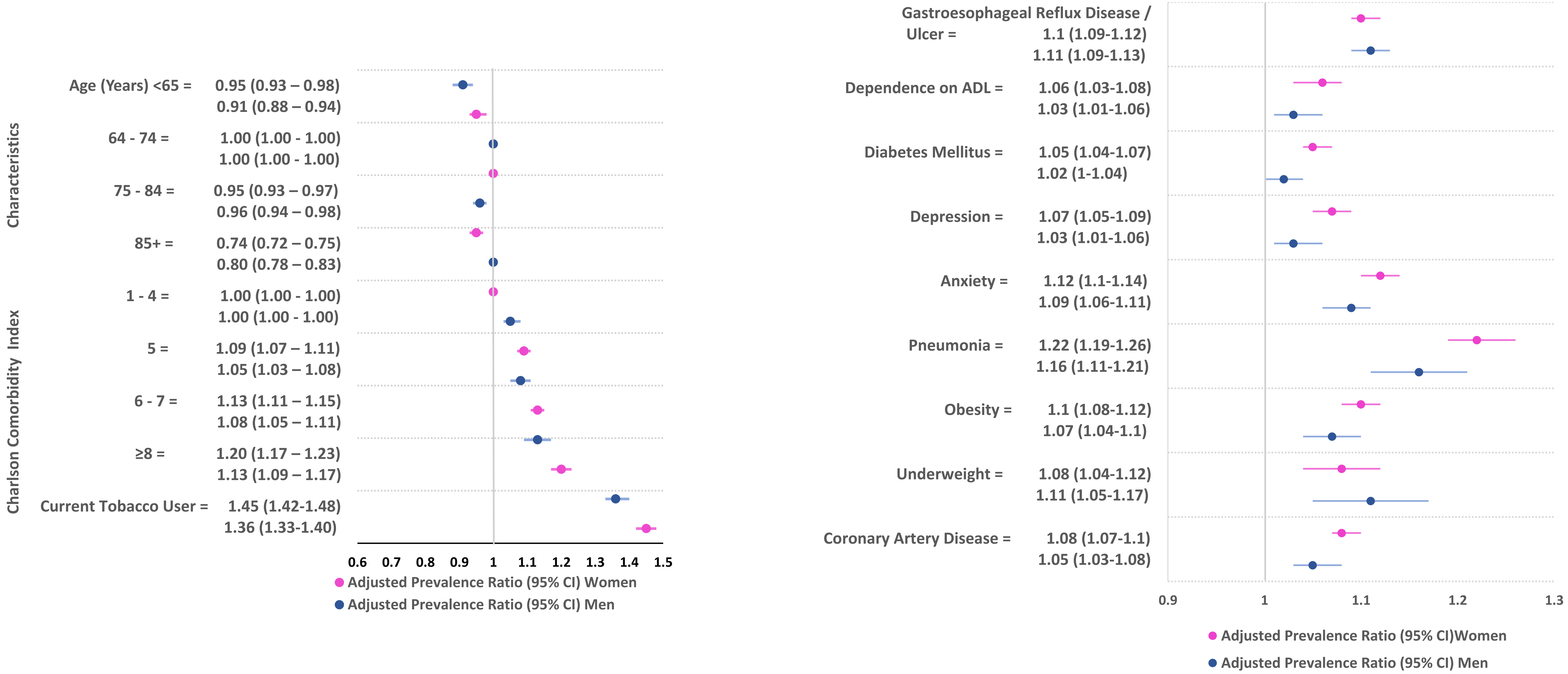

**Supplementary Figure 2: Association between sociodemographic, clinical characteristics and comorbid conditions with COPD, stratified by heart failure type**

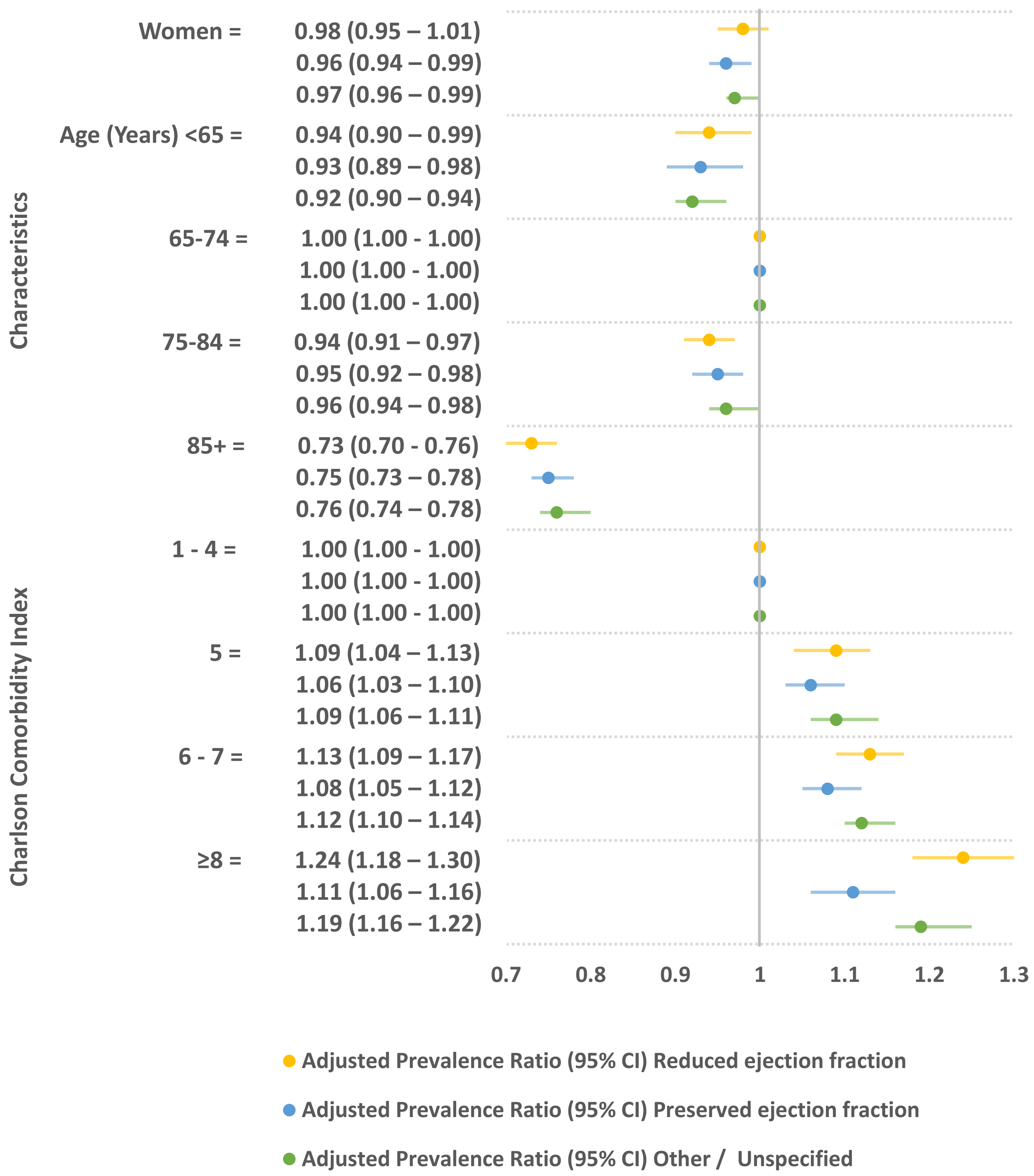


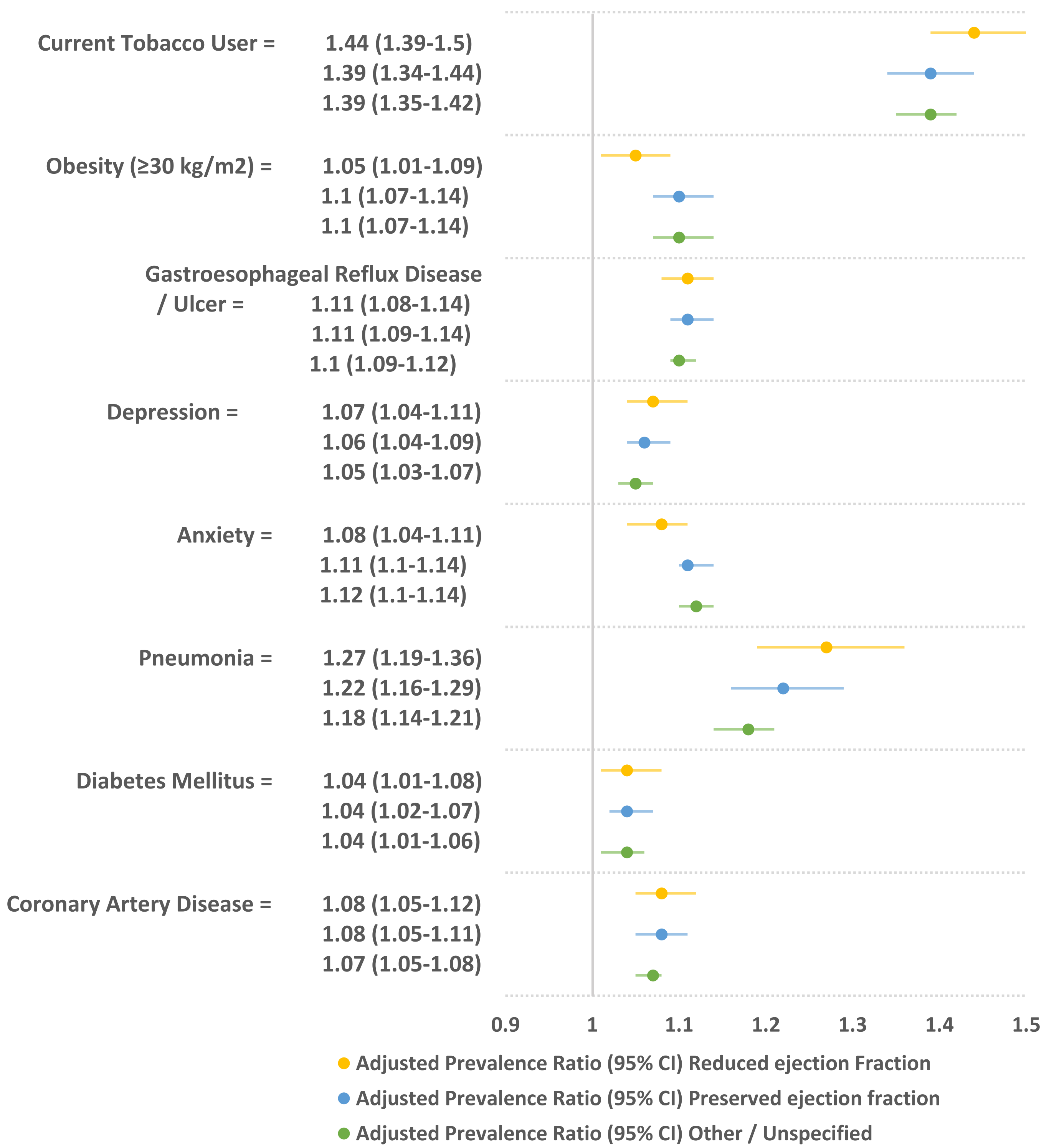

Supplement: Supplementary file 1 — Figure S1. Association between sociodemographic, clinical characteristics and comorbid conditions and COPD, stratified by sex. Figure S2: Association between sociodemographic, clinical characteristics and comorbid conditions with COPD, stratified by heart failure type. [file CRJ-17-1130-s001.pdf]
